# Barriers to social participation among patients with hemophilia: a qualitative study using social-ecological systems theory

**DOI:** 10.3389/fpubh.2026.1937214

**Published:** 2026-09-21

**Authors:** Jing Zhang, Menghan Xue, Xuewen Song, Yanyan Qu

**Affiliations:** Department of Hematology, Henan Cancer Hospital, Zhengzhou, China

**Keywords:** hemophilia, nursing, qualitative research, social participation, social-ecological systems theory

## Abstract

**Background:**

Social participation greatly determines health-related quality of life, while people with hemophilia face severe social engagement restrictions. Current studies only explore such barriers from single dimensions including joint function, employment and mental distress, lacking a comprehensive social-ecological perspective. Based on social-ecological systems theory, this study systematically analyzes multi-level barriers to social participation among Chinese hemophilia patients to support integrated nursing intervention development.

**Methods:**

A descriptive phenomenological qualitative design was employed. Purposive and snowball sampling strategies were used to recruit 15 adults with hemophilia from the hematology department of a tertiary hospital in China between February and July 2026. Individual semi-structured in-depth interviews were conducted using a topic guide informed by the three-system framework (micro-, meso-, and macro-). Data were analyzed using Colaizzi's seven-step method, with two researchers independently coding and reaching consensus through discussion. NVivo 14 was used to facilitate data management.

**Results:**

Nine themes and 20 sub-themes were identified across the three system levels. Micro-system barriers comprised: (1) progressive joint damage and functional limitations; (2) psychosocial distress and maladaptive coping; and (3) inadequate disease knowledge and self-management deficits. Meso-system barriers comprised: (4) catastrophic medication costs and family resource depletion; (5) family caregiving deficits and role overload; and (6) employment exclusion and social network contraction. Macro-system barriers comprised: (7) insurance coverage gaps and treatment access barriers; (8) geographic disparities in healthcare access; and (9) social stigma and inadequate institutional support. Critically, barriers across the three levels did not operate in isolation but interacted to produce a cumulative, compounding deprivation of social participation.

**Conclusions:**

Social participation barriers among people with hemophilia represent a complex, multi-layered phenomenon embedded within the micro-, meso-, and macro-systems of the social ecology. Nursing care should shift from a biomedical-only paradigm toward an integrated model: strengthening symptom management and psychological empowerment at the micro-level, promoting family enablement and social network reconstruction at the meso-level, and advancing policy advocacy and public education at the macro-level.

## Background

Hemophilia is an X-linked recessive inherited bleeding disorder caused by deficiency or dysfunction of coagulation factor VIII (hemophilia A) or coagulation factor IX (haemophilia B), clinically characterized by spontaneous bleeding, prolonged hemorrhage after trauma, and recurrent haemarthrosis predominantly affecting weight-bearing joints (1). According to the World Federation of Hemophilia (WFH) Annual Global Survey 2024, an estimated 1.125 million individuals worldwide are affected by haemophilia, with approximately 42,000 patients registered in China, although the true prevalence is likely to be substantially higher (2). With the widespread adoption of clotting factor replacement therapy and the advancement of comprehensive care models, life expectancy for people with hemophilia has progressively converged with that of the general population. Consequently, the focus of disease management has shifted from survival alone toward the enhancement of quality of life and the achievement of full social participation (3).

Social participation, broadly conceptualized as an individual's involvement in activities that provide interaction with others in society or the community, is recognized as a core indicator of social functioning and health-related quality of life (4). A growing body of evidence demonstrates that the level of social participation among people with hemophilia is significantly lower than that of the general population. Recurrent haemarthrosis leads to progressive joint deformity and functional impairment, severely restricting mobility and the range of social activities patients can undertake (5). Disease-related pain, fatigue, and the inherently unpredictable nature of bleeding episodes render it difficult for patients to maintain stable employment and social relationships, undermining the continuity of their social engagement (6). Furthermore, fear of disease-related stigma drives patients to actively avoid social interactions, resulting in self-imposed social withdrawal (7).

To date, research on social participation in hemophilia has predominantly examined isolated dimensions—including joint function and physical activity limitations (5), employment difficulties (8), and psychological distress (9)—without adopting a theoretical lens that situates patients within their broader social-ecological environment. Social-ecological systems theory, originally advanced by Bronfenbrenner in 1979 and subsequently refined, provides a powerful analytical framework for understanding the multi-level, interactive, and cumulative nature of social participation barriers (10). The theory posits that individual development is embedded within a set of nested, mutually interacting environmental systems. Specifically, three types of systems are distinguished: the micro-system, which encompasses the individual-level biological, psychological, and social subsystems that directly shape behavior; the meso-system, comprising the small-scale groups—family, work groups, and other social collectives—that exert influence on the individual; and the macro-system, referring to larger structures including organizations, institutions, communities, and sociocultural norms (11). Bidirectional interactions characterize the relationships across all three system levels, collectively shaping the trajectory and quality of an individual's social participation.

Applying social-ecological systems theory to the study of social participation in hemophilia makes it possible to transcend the fragmented approach of prior research and to systematically examine participation barriers at the micro- (individual bio-psychological), meso- (family–work–community), and macro- (healthcare system–institutional–cultural) levels, together with the mechanisms through which they interact. Accordingly, this study was guided by the following research questions: (1) What multi-level barriers to social participation do people with hemophilia experience at the micro-, meso-, and macro-system levels? (2) How do barriers across different system levels interact to produce cumulative effects on social participation? (3) What are the implications of these multi-level barriers for nursing practice and intervention design? To address these questions, a descriptive phenomenological qualitative approach, guided by social-ecological systems theory, was adopted to explore, from a nursing perspective, the multi-layered barriers to social participation experienced by people with hemophilia. It is anticipated that the findings will provide an empirical basis for developing integrative nursing intervention strategies grounded in the social-ecological framework.

## Methods

### Study design

A descriptive phenomenological qualitative design was adopted. Phenomenological inquiry seeks to understand the meaning and essence of lived human experience by returning ‘to the things themselves,' systematically describing and presenting the life-world of study participants through the process of bracketing—that is, setting aside the researcher's preconceptions (12). The present study followed Husserl's descriptive phenomenological tradition, employing Colaizzi's seven-step method as the specific analytical procedure. Reporting of this study was guided by the Consolidated Criteria for Reporting Qualitative Research (COREQ) 32-item checklist (13). A completed COREQ checklist is provided as Tables 1 and 2.

**Table 1:** Characteristics of study participants (*n* = 15).

ID	Age (years)	Type	Education	Residence	Marital status	Employment status	Family history	Severity	Age at first bleed	Age at diagnosis	Diagnostic delay (years)	Joint damage	Inhibitor
P1	27	A	High school	Rural	Unmarried	Employed	Negative	Severe	8 months	5 years	4	Knee, elbow, ankle	Negative
P2	35	A	Primary school	Rural	Unmarried	Unemployed	Positive	Severe	5 years	19 years	14	Knee, elbow	Negative
P3	31	B	High school	Rural	Married	Unemployed	Positive	Severe	< 1 year	15 years	14	Hip, knee	Positive
P4	68	A	High school	Rural	Married	Employed	Positive	Severe	< 1 year	14 years	14	Knee, elbow, ankle, hip, shoulder	Positive
P5	30	A	High school	Rural	Married	Employed	Negative	Severe	1 year	15 years	14	Knee, ankle	Negative
P6	17	A	No formal education	Urban	Unmarried	Unemployed	Negative	Severe	< 1 month	< 1 month	0	None	Positive
P7	27	B	Vocational	Urban	Married	Employed	Positive	Moderate	7-8 years	7-8 years	0	Knee	Negative
P8	34	B	College	Rural	Unmarried	Unemployed	Negative	Severe	< 1 month	6-7 years	6	Both knees	Negative
P9	56	A	Vocational	Rural	Married	Employed	Positive	Mild	5-7 years	19 years	12	Both knees	Negative
P10	24	A	College	Rural	Unmarried	Employed	Negative	Moderate	< 1 year	8 years	7	Knee, ankle	Negative
P11	51	A	Primary school	Rural	Unmarried	Unemployed	Positive	Severe	3-4 years	23 years	20	Knee, elbow, ankle, hip	Negative
P12	18	A	Vocational	Rural	Unmarried	Unemployed	Negative	Moderate	1-2 years	1-2 years	0	Knee, ankle	Positive
P13	33	A	College	Rural	Unmarried	Employed	Positive	Severe	5-6 years	21 years	15	Ankle, knee	Positive
P14	62	A	High school	Rural	Married	Unemployed	Positive	Severe	< 1 year	20 years	20	Knee, elbow, ankle, hip, shoulder	Positive
P15	41	A	Primary school	Rural	Married	Employed	Negative	Severe	< 1 year	5 years	5	Knee	Positive

Age at first bleed and age at diagnosis are presented as actual values or age ranges. Diagnostic delay = age at diagnosis - age at first bleed.

**Table 2:** Overview of themes and sub-themes identified through thematic analysis.

System level	Theme	Sub-theme
Micro	1. Progressive joint damage and functional limitations	1a. Irreversible joint damage from delayed treatment 1b. Acute bleeding episodes and life-threatening complications
Micro	2. Psychosocial distress and maladaptive coping	2a. Internalised stigma and illness concealment 2b. Chronic pain-related exhaustion and social withdrawal
Micro	3. Inadequate disease knowledge and self-management deficits	3a. Disease knowledge gaps and treatment non-adherence 3b. Unsafe self-administration without professional training
Meso	4. Catastrophic medication costs and family Resource depletion	4a. Medication costs consuming household income 4b. Loss of primary breadwinner and economic collapse
Meso	5. Family caregiving deficits and role overload	5a. Family knowledge deficits and harmful caregiving 5b. Caregiving voids and unsupported daily functioning 5c. Anticipatory anxiety and activity restriction
Meso	6. Employment exclusion and social network contraction	6a. Occupational marginalisation in low-wage employment 6b. Dual-pathway blockage of education and employment 6c. Progressive social network attrition
Macro	7. Insurance coverage gaps and treatment access barriers	7a. Insurance ceilings and inadequate prophylactic treatment 7b. Uniform reimbursement rules and structural inequity
Macro	8. Geographic disparities in healthcare access	8a. Primary care knowledge gaps and diagnostic Delays 8b. Centralised specialist services and medical travel burden
Macro	9. Social stigma and inadequate institutional support	9a. Public awareness deficits and forced illness concealment 9b. Insufficient welfare provision and persistent poverty

Themes are numbered 1-9; sub-themes are numbered with letter suffixes (e.g., 1a, 1b). System levels: Micro (individual), Meso (family/community), Macro (health system/policy/society).

### Setting and participants

Participants were recruited from the hematology department of a tertiary general hospital in Henan Province, China between February and July 2026 using a three-stage sampling strategy. First, purposive sampling was employed as the primary recruitment method. Eligible patients were initially identified by the first author through review of hematology department inpatient records during their hospitalization. The first author approached potential participants face-to-face during their inpatient stay, explained the study purpose and procedures, and provided a written information sheet. For participants under 18 years of age (P6, P12), written informed consent was obtained from a parent or guardian, and the adolescent provided assent. Second, maximum variation sampling was applied to ensure sample heterogeneity across the following dimensions: age, disease type, disease severity, educational attainment, residence, employment status, and marital status. Third, snowball sampling complemented the initial purposive sampling. During the consent process, participants were informed that they could refer other individuals with hemophilia who might be willing to participate. When a participant provided a referral, the research team contacted the referred individual directly to explain the study and assess eligibility. Of the 15 participants, 13 were recruited through purposive sampling and 2 through snowball sampling.

The inclusion criteria were: (1) confirmed diagnosis of hemophilia A or B in accordance with the Chinese Expert Consensus on the Diagnosis and Treatment of Hemophilia (2017 edition) (14); (2) aged 16 years or older; (3) duration since confirmed diagnosis of at least 6 months; (4) alert and oriented, without communication impairments; and (5) voluntary participation with provision of written informed consent. Exclusion criteria were: (1) presence of severe mental disorders or cognitive impairment; (2) co-existing severe illnesses that could independently and substantially affect social participation (e.g., end-stage malignancy); and (3) being in an acute bleeding episode or critically ill at the time of recruitment.

The sample size was determined by the principle of data saturation. After each interview, the research team performed preliminary coding and assessed whether new themes or sub-themes had emerged. Following the 12th interview, the team conducted an interim analysis of all coded data and identified that the nine themes and 20 sub-themes had substantially stabilized. Three subsequent interviews (P13-P15) were then conducted to test for saturation; during these interviews, no new themes or sub-themes emerged, and participant narratives confirmed and enriched existing themes without introducing novel conceptual elements. Data saturation was therefore deemed to have been reached after interviewing 13 participants. Two additional participants (P14-P15) were retained in the final sample to corroborate the findings, yielding a total of 15 participants. All participants were male, ranging in age from 17 to 68 years (median 33 years). Twelve participants had hemophilia A and three had hemophilia B. Disease severity was classified as severe in 11 cases, moderate in three, and mild in one. Educational attainment ranged from primary school to undergraduate level. Occupational status included employed or unemployed. All participants were assigned anonymous codes from P1 to P15. Their general characteristics are presented in Table 1. The study received ethical approval from the institutional review board of the study hospital (Ethics Approval No.: 2026-311).

### Data collection

Data were collected primarily through individual semi-structured in-depth interviews. An interview topic guide was developed by the research team based on the social-ecological systems theory and refined through literature review and expert consultation. The guide was piloted with two participants, after which minor revisions were made to improve clarity and flow. The final topic guide comprised 12 open-ended questions across three system levels. At the micro-system level, questions explored experiences of pain, bleeding, and joint impairment; psychological responses to the diagnosis; the nature and extent of daily activity restrictions; and typical daily routines. At the meso-system level, questions examined family attitudes and support, shifts in family roles, employment and educational experiences, and changes in social networks. At the macro-system level, questions investigated difficulties in accessing healthcare, the financial burden of medication and health insurance coverage, public awareness of hemophilia, and experiences of discrimination or social exclusion.

All interviews were conducted by the first author, who has over 10 years of clinical nursing experience in hematology and had received formal training in qualitative research methods. Each participant was interviewed between one and three times, with individual sessions lasting 30–60 min (mean duration 46 min). All interviews were conducted in a private setting (a hospital teaching room, the participant's bedside, or a private consultation room), and no family members, nurses, or other third parties were present during the interviews unless the participant explicitly requested company for safety or comfort reasons. All interviews were audio-recorded with participants' informed consent, and field notes were taken simultaneously to document non-verbal cues including facial expressions, tone of voice, pauses, and body language. Each interview commenced with an open-ended introductory question, after which the topic guide was used to progressively explore participants' experiences across the three system levels. Throughout the interviews, open-ended and non-leading prompts were used to allow participants' narratives to unfold naturally.

### Data analysis

Interview data were analyzed using Colaizzi's seven-step method (15), which comprised the following steps: (1) reading and re-reading all interview transcripts in their entirety to acquire a sense of the whole; (2) extracting significant statements directly pertinent to the phenomenon under investigation; (3) formulating meanings from each significant statement; (4) clustering the formulated meanings into theme clusters; (5) integrating the theme clusters into an exhaustive description of the phenomenon; (6) condensing the exhaustive description into the fundamental structure of the phenomenon; and (7) returning to participants to validate the findings through member checking. Data management and coding were facilitated using NVivo 14 software (QSR International Pty Ltd, Melbourne, Australia). Two researchers independently coded all transcripts, and the inter-coder agreement rate was 91.7% (Cohen's κ = 0.87). All coding discrepancies were resolved through discussion until consensus was reached; where consensus could not be reached, a third researcher served as adjudicator. To maintain a comprehensive audit trail, the following documentation was maintained throughout the analysis: (1) coding decision logs in NVivo 14, recording the rationale for each code assignment and any modifications made during iterative analysis; (2) coding memos documenting the evolution of codes from initial open codes to focused codes and final themes, including the rationale for code aggregation and theme development; (3) consensus meeting minutes recording the specific coding discrepancies discussed, the resolution process, and the final decisions; and (4) a thematic analysis log documenting the step-by-step progression through Colaizzi's seven-step method, including the transition from significant statements to formulated meanings to theme clusters. All analytical documentation is retained and available for audit purposes.

### Reflexivity

The first author, who conducted all interviews, is a registered nurse with over 10 years of clinical experience in hematology nursing. The corresponding author (MX) is a master's-prepared nursing researcher; XS and QY are registered nurses with clinical hematology experience. This clinical background provided her with in-depth knowledge of hemophilia and its management, facilitating rapport-building with participants and enabling probing questions that drew out clinically relevant experiences. However, the research team acknowledged that this background could also introduce potential biases: (1) clinical familiarity might lead the interviewer to interpret participants' narratives through a biomedical lens, potentially overlooking the social and experiential dimensions of their accounts; (2) prior clinical relationships with some participants (who were former or current patients) might influence the depth of disclosure; and (3) the interviewer's advocacy for improved haemophilia care might unconsciously steer narratives toward systemic deficiencies. To manage these potential biases, several reflexive strategies were employed throughout the study. First, before commencing fieldwork, the first author engaged in a bracketing exercise, documenting her preconceptions about hemophilia patients' social participation in a reflexive journal. Second, immediately after each interview, she completed a reflexive memo documenting her emotional responses, potential biases, and any assumptions that may have influenced the interview conduct or initial interpretation. Third, regular peer debriefing sessions were held with the research team, during which the first author's interpretations were challenged and alternative readings were explored. Fourth, during data analysis, coded transcripts and emerging themes were reviewed by the second author, who does not have a clinical hematology background, providing an external perspective that helped identify interpretations potentially influenced by clinical assumptions.

### Strategies to ensure rigor

Several strategies were employed to ensure the trustworthiness of the study with respect to credibility, transferability, dependability, and confirmability (16). Credibility was established through triangulation of data sources (recruiting a diverse sample via maximum variation purposive sampling) and member checking, whereby the investigator-derived themes, sub-themes, interpretations, and conclusions were shared with and confirmed by interested participants. To enhance transferability, a comprehensive description of the study setting, participant characteristics, and their lived experiences of social participation barriers was obtained, enabling comparisons across different settings and contexts. Dependability was addressed by providing a detailed, transparent account of all research steps—from study design through to data collection and analysis—allowing future researchers to follow the process. To ensure confirmability, the researchers maintained a reflexive stance throughout data collection and analysis, reflecting on how their own assumptions and clinical experiences might influence the interpretation of data. For example, after each interview, the interviewer reflected on whether her unconscious responses during the interaction had influenced the participant's expression.

## Results

Through iterative coding and constant comparative analysis of the interview data from 15 participants, and guided by the three-system framework of the social-ecological systems theory, a total of three thematic domains (micro-system barriers, meso-system barriers, and macro-system barriers), nine themes, and 20 sub-themes were identified. Each theme was found to be directly associated with impairments in social participation. An overview of the themes and sub-themes across the three system levels is presented in Table 2.

### Micro-system barriers

The micro-system refers to the individual system and encompasses the biological, psychological, and social subsystems that directly influence behavior. This level focuses on the direct impact of disease-induced physical impairment, psychological deterioration, and deficits in disease self-management on social participation. Collectively, the interviews with all 15 participants revealed a micro-system chain of social participation barriers extending from physical functional damage, through accumulation of psychological burden, to impediments in self-care.

#### Theme 1: progressive joint damage and functional limitations

This theme captures the direct restrictions on social participation resulting from progressive physical deterioration due to recurrent joint hemorrhages, as reported by all 15 participants. Bleeding triggers were diverse—including physical exertion, spontaneous onset, and seasonal transitions—and joint damage followed a gradient of increasing severity from activity limitations to multi-joint replacement to complete disability. Acute haemorrhagic events could result in prolonged bed rest or even life-threatening emergencies, continuously compressing patients' activity range and social participation opportunities.

##### Sub-theme 1a: Irreversible joint damage from delayed treatment

This sub-theme addresses the diversity and unpredictability of bleeding triggers, as well as the irreversible joint damage and activity limitations arising from diagnostic delays and recurrent hemorrhage, which effectively severed patients' pathways to daily social activities. Physical exertion, prolonged standing, accidental falls, and apparently spontaneous episodes all triggered acute joint bleeds. Diagnostic delays of years to decades were common; the longer the delay, the more irreversible the joint damage. Joints afflicted by chronic recurrent hemorrhage developed deformity and functional impairment, and even those receiving joint replacement surgery did not always recover satisfactorily, with social participation capacity declining correspondingly.

“*When I was six or seven years old, the bleeding was quite frequent, and gradually my knee became locked.”* (P1)

“*I had knee replacement surgery in 2022, but the joint didn't really improve. When the pain flares up, I still can't walk.”* (P4)

“*If I overdo the physical activity, bleeding follows. When the seasons change between winter and summer, when I'm overtired, or when I stay up late—I bleed. Now both my knees are deformed; I can only bend them to less than ninety degrees.”* (P9)

##### Sub-theme 1b: acute bleeding episodes and life-threatening complications

This sub-theme addresses the acute consequences of haemorrhagic events for social participation, which were completely disrupted. After each episode of bleeding, patients were compelled to remain in bed for extended periods, during which all activities—study, work, and socializing—came to a standstill. Several participants had also experienced sudden major bleeds that directly threatened their lives. Haemorrhagic events thus inflicted not only long-term joint damage but also mandatory, recurrent interruptions to social participation during the acute phase itself.

“*Before I was diagnosed, I was bleeding three or four times a month on average. After a severe bleed, I'd be bedridden at home for over a month before recovering.”* (P3)

“*Twice in my life I had severe haematomas in my throat—blood clots blocking my airway so I couldn't breathe.”* (P4)

“*In 2014 I had a nosebleed that wouldn't stop for a whole month. They said if it had gone on a few more hours, I wouldn't have made it.”* (P5)

#### Theme 2: psychosocial distress and maladaptive coping

This theme covers the persistent psychological impediments to social participation arising from long-term illness and social exclusion, reported by all 15 participants. Psychological damage accumulated across the disease course, manifesting as the internal psychological mechanism underlying patients' active withdrawal from social participation.

##### Sub-theme 2a: internalized stigma and illness concealment

This sub-theme addresses how patients, having internalized a sense of inferiority from childhood due to physical differences, chose to conceal their diagnosis in adulthood and ultimately retreated into self-imposed social isolation, voluntarily relinquishing social participation. To varying degrees, all 15 participants described concealing their condition from others.

“*It's like being inside a box… no friends, never been in a relationship, afraid that no one would understand.”* (P1)

“*I never dared to tell anyone from when I was small. Better not to cause trouble.”* (P6)

“*If I didn't have this disease, at least my life would be roughly similar to other people's. Now it's as if I'm one step below everyone else.”* (P15)

##### Sub-theme 2b: chronic pain-related exhaustion and social withdrawal

This sub-theme addresses the compounding effect of recurring pain, persistent employment obstacles, progressive social withdrawal, and bereavement—multiple factors overlapping to create a state of profound physical and psychological depletion, further eroding patients' will and capacity to sustain social participation.

“*The psychological suffering is harder to bear than the physical pain and the financial pressure.”* (P6)

“*I keep thinking—might I die suddenly one night too? I can sleep the first half of the night, but the second half, I can't.”* (P2)

“*Just take it one day at a time.”* (P8)

#### Theme 3: inadequate disease knowledge and self-management deficits

This theme addresses the indirect effect of inadequate disease knowledge and weak self-management skills on social participation, reported by all 15 participants. Knowledge deficits and lack of self-care competence constituted one of the micro-system barriers; unregulated self-care perpetuated a persistent bleeding risk that indirectly compelled patients to further avoid social participation activities.

##### Sub-theme 3a: disease knowledge gaps and treatment non-adherence

This sub-theme identifies patients' deficits in core knowledge about hemophilia—including etiology, prophylactic treatment, and novel therapies—as well as harmful misconceptions. All 15 participants exhibited varying degrees of knowledge deficiency. These knowledge gaps prevented effective management of bleeding risk, forcing patients to further restrict their own activities as a protective strategy.

“*I don't really understand it either… no management at all—no prophylaxis, no fixed dosage, no proactive learning.”* (P14)

“*After my diagnosis, I didn't understand any of this. I didn't know how to treat it. The doctor just said, come to hospital when you bleed and we'll give you a transfusion—no other option was offered.”* (P13)

“*I never actively tried to learn about it, and I had no idea how to prevent it.”* (P11)

##### Sub-theme 3b: unsafe self-administration without professional training

This sub-theme describes patients' process of learning intravenous injection and routine self-care without guidance from healthcare professionals. Failed injections and complication risks discouraged patients from venturing out for social activities. The learning pathway of all 15 participants was idiosyncratic, with no standardized instruction.

“*Three attempts on the left hand, three on the right… six failed attempts altogether. After drying my hand, I kept trying—the seventh attempt got it.”* (P5)

“*Tried twice, couldn't hit the vein, couldn't bring myself to go in. Kept asking the village doctor to do it.”* (P14)

“*I learned the basics of sterilization from my high school biology and chemistry lab… watched how others did it, mimicked them, and after a few days I got it.”* (P13)

### Meso-system barriers

The meso-system refers to small-scale groups that exert influence on the individual system, including family, work groups, and other social collectives. This level focuses on how interactive barriers between patients and their families, workplaces, and communities further restrict the space available for social participation. Collectively, the interviews revealed a meso-system chain of barriers extending from depletion of economic resources, through deficits in caregiving support, to simultaneous attrition of employment and social interaction.

#### Theme 4: catastrophic medication costs and family resource depletion

This theme reveals the cumulative burden of hemophilia-related medical costs on family finances and the consequent constraint on social participation, reported by all 15 participants. Severe depletion of economic resources rendered patients unable to sustain the basic material conditions required for social participation, generating a chain effect: high medication costs → family impoverishment → loss of social participation capacity.

##### Sub-theme 4a: medication costs consuming household income

This sub-theme identifies the disproportionately high share of haemophilia medication costs within household expenditure, which severely compressed the material conditions necessary for normal social life. Across different life stages, all 15 participants confirmed this predicament: beyond medication costs, all other living expenses were drastically reduced, with resources for socializing, recreation, and education approaching zero.

“*Medical costs per year… at the very minimum, that's probably eighty percent of the total household income.”* (P15)

“*According to my weight, the dosage isn't enough… but the insurance ceiling is limited, so I don't dare take more.”* (P2)

“*Five vials of factor IX cost 3,800 yuan. Even after reimbursement, the co-payment still represents enormous pressure for families without a stable income.”* (P3)

##### Sub-theme 4b: loss of primary breadwinner and economic collapse

This sub-theme identifies hemophilia households' near-universal dependence on a single income source. With only a thin economic margin, any misfortune befalling the patient or the primary caregiver immediately severed the household's economic chain, eliminating the material foundation upon which social participation depended.

“*I'm counting on my old father to earn money and support the whole family… his wage supports the whole extended family.”* (P9)

“*All the income depends entirely on yourself alone… four people, all depending on you. Per capita income is less than 2,000 yuan.”* (P15)

“*After my father died, the economic pillar of the family was gone.”* (P13)

#### Theme 5: family caregiving deficits and role overload

This theme encompasses the indirect constraints on social participation arising from insufficient family caregiving support, reported by all 15 participants. Family functions as a critical support structure for patients' engagement with society; deficiencies in family caregiving directly weakened the rearguard protection necessary for patients to participate in social activities, leaving them isolated and unsupported.

##### Sub-theme 5a: family knowledge deficits and harmful caregiving

This sub-theme identifies caregiving deviations arising from family members' inadequate understanding of hemophilia, ranging from incorrect folk remedies to conditional emotional support to information gatekeeping. Incorrect caregiving not only worsened the patient's condition but also reduced the physical capital available for social participation.

“*His mother didn't know any better—she applied hot water to the bleeding joint, rubbed salt water on it to bring down the swelling. [This directly led to permanent knee joint deformity.]”* (P9)

“*She (my wife) doesn't really understand any of this… she just sees it as a burden.”* (P15)

“*The doctor never communicated the diagnosis directly with me—only with my parents.”* (P6)

##### Sub-theme 5b: caregiving voids and unsupported daily functioning

This sub-theme identifies caregiving voids arising from parental separation, labor migration, advanced age and illness, or bereavement, which left patients unable either to safely engage in social activities or to receive timely assistance after hemorrhage.

“*Dad's not home, and my grandmother can't manage. She's so old that it's more like I'm looking after her.”* (P1)

“*My mother is 95 and has dementia. My brothers are all struggling themselves—there's simply no help available.”* (P11)

“*Injecting myself, going to hospital—all alone, no family to help.”* (P5)

##### Sub-theme 5c: anticipatory anxiety and activity restriction

This sub-theme identifies a deep-seated fear of complete family collapse should the patient become fully dependent—a fear particularly pronounced among middle-aged and older participants. This anticipatory anxiety discouraged patients from attempting any social participation activity that might entail risk.

“*I need to take good care of the person who supports me, and arrange things for the people I need to support… If I collapse, what do we do?”* (P9)

“*Once you go down, it's all over. Who's going to look after you? And you're still the main pillar of the household.”* (P15)

#### Theme 6: employment exclusion and social network contraction

This theme encompasses the comprehensive employment and social difficulties arising from the dual effect of physical limitations and financial pressure, revealing the simultaneous multi-dimensional compression of social participation space, reported by all 15 participants. Employment represents one of the most important channels of social participation for adults; the social network is the central vehicle of social engagement; and the simultaneous attrition of both means that patients' social participation is rendered almost entirely inaccessible.

##### Sub-theme 6a: occupational marginalization in low-wage employment

This sub-theme identifies the exclusion and marginalization of hemophilia patients in the labor market: employment is highly concentrated in low-threshold, low-income, unprotected types of work, making it impossible for patients to achieve social integration and a sense of value through normal labor. Among the 15 participants, occupational types included e-commerce operations (desk-based), reassignment from a steel production line to the telephone dispatch room, assisting at a friend's teahouse, and self-employed barbershop barely covering basic expenses.

“*I can't take orders from others… when you're your own boss, at least you know your own limits.”* (P15)

“*I work in e-commerce operations—sitting there, not much physical movement.”* (P7)

“*With this disease, there's no stable job. I survive on minimum social allowance and casual work.”* (P8)

##### Sub-theme 6b: dual-pathway blockage of education and employment

This sub-theme describes how disease-related interruption of education, followed by rejection from the labor market due to physical limitations, simultaneously severed the two primary pathways into social participation—education and employment—creating a structural predicament of ‘disease → school dropout → unemployment → absence of social participation.'

“*After the New Year, they told me not to come back… because of this disease.”* (P2)

“*After I started working at the factory, my knee bled within three days and they let me go. If you tell them about the disease upfront, they won't let you in at all.”* (P3)

“*I had barely been at school for two months each semester… I dropped out in the second year of junior high school. That was my own decision.”* (P5)

##### Sub-theme 6c: progressive social network attrition

This sub-theme addresses how patients, unable to travel far due to physical limitations and with an ever-narrowing range of activities, progressively lost connection with the outside world. Social participation declined from a quantitative reduction to a qualitative absence. All 15 participants exhibited a markedly sparse life trajectory.

“*No friends on QQ or WeChat. Never made any friends.”* (P1)

“*I rarely go out—just keeping to your own shop… if someone treats you once, you'd need to treat them back… I can't physically handle that.”* (P15)

“*I don't dare go far from school… walk too far and I'm worried that more activity will cause another bleed.”* (P9)

### Macro-system barriers

The macro-system refers to larger systems beyond small-scale groups, including organizations, institutions, communities, and socio-cultural forces. This level focuses on how macro-level factors—healthcare insurance policies, primary care resource allocation, social cognition, and public welfare—systematically obstruct patients' channels of social participation from institutional and cultural dimensions. The experiences of all 15 participants, spanning from a time when no insurance existed to the present day when insurance exists but remains far from adequate, collectively revealed the structural deprivation of social participation imposed by the macro-system.

#### Theme 7: insurance coverage gaps and treatment access barriers

This theme covers the institutional constraints on social participation arising from healthcare insurance system inadequacies, reported by all 15 participants. From full out-of-pocket costs in earlier years and the prolonged absence of hemophilia medications from the insurance formulary, to the current reimbursement ceilings that fail to reflect individual clinical needs, multiple policy deficiencies have prevented patients from accessing adequate treatment and thus rendered them unable to maintain the basic physical condition required for social participation.

##### Sub-theme 7a: insurance ceilings and inadequate prophylactic treatment

This sub-theme identifies the inability of current insurance reimbursement ceilings to meet the medication requirements of evidence-based prophylactic therapy. The absence of adequate treatment directly results in persistently high bleeding frequency, progressive joint damage, and a corresponding decline in social participation capacity. All 15 participants provided evidence across different periods: during the era of full out-of-pocket costs, treatment was simply unaffordable; even after medications were included in insurance formularies, forced dose reductions or abandonment of prophylaxis persisted due to ceiling restrictions.

“*One hundred and fifty thousand yuan per year isn't enough—I have to borrow. I borrow, repay, and then it's still not enough, so I borrow again.”* (P11)

“*There's a drug shortage right now… patients are lending medications to each other among ourselves… we'll pay back when next year's quota comes through.”* (P14)

“*I can only purchase thirty bypass therapy vials per year. One bleed uses five vials, so the quota runs out very quickly.”* (P4)

##### Sub-theme 7b: uniform reimbursement rules and structural inequity

This sub-theme identifies that insurance reimbursement ceilings do not differentiate by disease severity, bleeding frequency, or body weight, applying a uniform standard regardless of individual need. This results in patients with the most frequent bleeds and the most severe social participation barriers receiving the least proportionate coverage—a structural inequity.

“*One fixed quota for everyone—some have enough, some are crushed by it… it's the severe cases that get the worst deal. The quota doesn't distinguish between severe and mild—one size fits all.”* (P15)

“*Severe patients bleed two or three times a week; the quota is simply not enough.”* (P9)

“*Thirty vials a year, quota exhausted very quickly.”* (P4)

#### Theme 8: geographic disparities in healthcare access

This theme encompasses the constraints on social participation arising from severely inadequate primary healthcare services, reported by all 15 participants. Weaknesses in primary care not only delayed diagnosis and evidence-based treatment but also compelled patients to expend enormous time and physical effort to access basic care, further compressing the resources available for social participation.

##### Sub-theme 8a: primary care knowledge gaps and diagnostic delays

This sub-theme identifies comprehensive deficiencies in diagnostic and treatment capabilities for hemophilia at the primary care level, including instances of iatrogenic harm from incorrect treatment. Prolonged diagnostic delays meant that patients failed to receive treatment during a critical window when joint protection would have been feasible; joint damage became irreversible before diagnosis was reached, permanently reducing the physical capital for social participation.

“*At the county hospital, the doctor doing the examination said, ‘I've never seen this disease before—you're giving me a hard problem.”'* (P14)

“*In our county hospital, they couldn't identify what it was… county-level hematology departments understand very little about this disease.”* (P13)

“*At the time, a lot of hospitals didn't know about this… they diagnosed me with arthritis, rheumatism… all sorts of things—acupuncture, massage—you name it, they tried it.”* (P5)

##### Sub-theme 8b: centralized specialist services and medical travel burden

This sub-theme describes how the extreme concentration of hemophilia specialist services in large urban centers compelled patients to undertake long-distance travel, which itself constituted a trigger for bleeding, trapping them in a vicious cycle of ‘traveling for treatment → bleeding → requiring further treatment.' Each hospital visit consumed social participation opportunities.

“*In the early years, there was completely no medication available locally, so we had to make the long journey to Zhengzhou, or even Beijing, to obtain drugs.”* (P6)

“*All the back-and-forth travel causes bleeding—joint bleeds… the round trip costs over a hundred yuan.”* (P15)

“*The back and forth causes bleeding. Three or four h' journey and my legs are already swollen.”* (P1)

#### Theme 9: social stigma and inadequate institutional support

This theme encompasses the deep-level impact on social participation of societal misconceptions and inadequate welfare provision—a dual macro-system challenge—reported by all 15 participants. Social misconceptions pushed patients out of the arena of social participation through mechanisms of exclusion, while inadequate welfare provision severed the last policy-mediated possibility of social participation through institutional support.

##### Sub-theme 9a: public awareness deficits and forced illness concealment

This sub-theme identifies the lack of awareness among the general public and institutions regarding hemophilia as a rare disease, and the resultant exclusion, misunderstanding, and discrimination that compelled patients to conceal their diagnosis as a strategy to avoid social rejection—at the cost of comprehensively compressing the scope and depth of their social participation.

“*There's a cultural custom in rural areas—people say that if word gets out about your illness, you won't even be able to find a wife.”* (P4)

“*There's not enough awareness—people still don't really know what hemophilia is. Ordinary people think we're just all-round serious cases.”* (P15)

“*Even the teachers and classmates didn't know.”* (P7)

##### Sub-theme 9b: insufficient welfare provision and persistent poverty

This sub-theme identifies that available government subsidy amounts are insufficient to cover the high treatment costs and basic living needs of hemophilia patients. When economic poverty and social exclusion overlap, the last policy safeguard for social participation is rendered ineffective.

“*The disability allowance is sixty yuan a month… the minimum living subsidy is two hundred and ten yuan a month… for this disease, it makes no difference.”* (P15)

“*Minimum subsidy plus new cooperative medical insurance, and we still survive on loans. You live according to what you have.”* (P14)

## Discussion

To our knowledge, this study is among the first to systematically apply social-ecological systems theory to explore the barriers to social participation among people with hemophilia in China. Our findings demonstrate that social participation barriers in this population are not confined to a single dimension but represent a complex, multi-level phenomenon embedded across micro-, meso-, and macro-system contexts. Critically, barriers at each level do not operate independently; rather, they interact across system levels to produce a cumulative, compounding deprivation of social participation.

### Micro-system level: from physical care to psychological empowerment to rebuild participation capacity

Our findings indicate that micro-system barriers are the most direct and proximal sources of social participation impairment among people with hemophilia. Irreversible joint damage from recurrent haemarthrosis not only restricted patients' mobility but also directly disrupted the continuity of their social participation through repeated episodes of acute-phase immobilization. This finding aligns with international evidence demonstrating significant negative correlations between bleeding frequency and both work productivity and social activity participation (5, 17). The present study extends this literature by revealing that, in the context of limited primary healthcare resources in China, diagnostic delays spanning years to decades permanently deprived patients of the physical foundation for full social participation while missing the critical window for early intervention. This suggests that nursing interventions should extend beyond the management of acute bleeding to incorporate prophylactic treatment education and joint protection into early care plans, thereby maximizing preservation of patients' social participation capacity.

Disease-related psychological burden emerged as a substantial barrier to social participation. The majority of participants had been aware of their physical difference from others since childhood and had chronically suppressed disclosure of their diagnosis and curtailed social activities—a finding echoed in research on other chronic hereditary conditions (17). Notably, the lived experiences of all 15 participants demonstrated that stigma permeated their entire life course: from the sense of confinement ‘like being inside a box' in childhood to the internalized self-appraisal of being ‘one step below everyone else' in adulthood. This diminished sense of self did not dissipate over time and continuously eroded patients' motivation to re-engage in social participation. This observation is consistent with a core tenet of social-ecological systems theory, namely, that bidirectional interactions exist between an individual's psychological state and their social participation behavior (10). From a nursing perspective, the promotion of social participation must simultaneously address psychological barriers. Specifically: first, targeted psychological interventions should guide patients in examining and reframing negative illness cognitions, alleviating the constraining effect of negative affect on willingness to engage in social activities; second, Acceptance and Commitment Therapy (ACT) can help patients accept the reality of their condition while reconstructing goals and actions for social participation; and third, a peer companionship mechanism—whereby experienced patients serve as participatory role models for the newly diagnosed—can help disrupt cycles of social isolation (18).

Our findings also revealed that knowledge deficits and inadequate self-care competence indirectly compressed patients' social activity space through persistently elevated bleeding risk as a mediating mechanism. The experiences described—from having no prophylactic strategy to succeeding only after seven injection attempts—reveal a systemic absence of structured hemophilia self-management education. The stronger patients' self-care competence, the greater their sense of control over bleeding risk, and the more their confidence and willingness to engage in social activities correspondingly increased. Chinese research has confirmed that caregivers' disease knowledge is closely associated with patients' safety outcomes and social participation quality (19). Enhancing patients' and family members' disease self-management capacity therefore constitutes an important micro-system nursing entry point for improving social participation.

### Meso-system level: family financial pressure and caregiving deficits constraining participation space

Our findings reveal that the catastrophic medication cost burden—universally experienced by all 15 families—represents one of the most pervasive meso-system barriers. Medication costs consuming at least 80% of household income severely compressed routine household expenditure, effectively stripping patients of the material basis for social participation. Many households depended on a single income source; the loss of the primary breadwinner represented not merely financial difficulty but the simultaneous collapse of dual support systems—caregiving and employment—underpinning the patient's social participation. This finding is consistent with Chinese research confirming that annual per-person medication expenditure for hemophilia patients exceeds CNY 46,000, and that medication costs alone have driven many families into financial hardship (20). International evidence similarly documents that even in high-income countries, out-of-pocket rare disease costs constitute a substantial proportion of household income; a Turkish study of families affected by rare diseases reported average annual out-of-pocket costs of approximately €2,877 (21). The cross-national compression of family social participation capacity by catastrophic medication expenditure thus represents a universal pattern.

Deficiencies in family caregiving support operated through three distinct mechanisms, each constraining social participation through different pathways. First, knowledge deficits: family members' incorrect caregiving not only exacerbated the patient's condition but also reduced the physical capital available for social participation. The case of P9, whose mother applied hot water to the bleeding site, directly causing permanent knee joint deformity, exemplifies how caregiving errors can irreversibly diminish social participation capacity. Second, structural caregiving voids arising from parental separation, labor migration, advanced age and illness, or bereavement left patients unable either to safely engage in social activities or to receive timely assistance after hemorrhage. Third, role pressure on family members who independently shouldered caregiving responsibilities also resulted in severe compression of their own social participation space. These findings are consistent with recent research confirming that hemophilia caregivers' disease knowledge and caregiving skills are broadly insufficient, representing a significant safety concern (19).

Employment difficulties constituted the most salient meso-system finding. Our analysis revealed that patients faced not merely unemployment but the simultaneous blockage of both primary social participation pathways—education and employment. International research confirms that unemployment rates among people with hemophilia are significantly higher than in the general population, and that bleeding frequency directly reduces work productivity and social activity participation (22); a multi-country European survey similarly found that unemployment rates among adult hemophilia patients far exceeded those of age-matched healthy individuals (23). Relative to international findings, the present study's identification of a ‘dual-pathway blockage' mechanism—whereby educational disruption precedes employment exclusion in a chain of sequential damage—is infrequently described as a distinct phenomenon in the non-Chinese literature. This likely reflects the reality that Chinese hemophilia patients experience disabling joint damage earlier in life and have more restricted educational opportunities (24). These findings suggest that healthcare professionals need to transcend the purely clinical role and proactively advocate for reasonable accommodations for patients in educational and workplace settings, functioning as practical advocates for patients' rights to social participation. Recent research further indicates that reducing stigma and enhancing social support are key pathways to broadening social participation among hemophilia patients (25).

### Macro-system level: structural barriers and the nursing profession's role in promoting social participation

Macro-system barriers constitute the deepest and most resistant structural roots of social participation impairment. The uniform insurance reimbursement ceiling—applied irrespective of disease severity—creates an institutional paradox: patients with the most severe disease and the greatest social participation barriers receive proportionally the least adequate insurance protection. P15′s observation that ‘one fixed quota for everyone—some have enough, some are crushed by it' captures the gulf between institutional design and actual need. The World Health Organization's International Classification of Functioning, Disability and Health (ICF) framework explicitly recognizes that an individual's social participation is determined not only by bodily function but also, and profoundly, by environmental factors including policy, institutional structures, attitudes, and the physical environment (26). Both domestic and international research confirms that inadequate insurance coverage for rare disease patients represents a significant macro-system factor impairing social participation (20, 21). Recent analyses indicate that China's current rare disease protection system remains insufficient in both depth and breadth of coverage, and recommend establishing a dedicated insurance mechanism for rare diseases to genuinely secure the economic prerequisite for patients' social participation (27).

The impact of primary healthcare weaknesses on social participation operates at two levels. First, diagnostic delays deny patients intervention during a period when joint protection was still achievable, permanently diminishing their physical capital for participation. Second, the burden of medical travel itself consumes enormous quantities of the time, physical energy, and economic resources that patients might otherwise invest in social participation—a tension crystallized by P15′s observation that ‘all the back-and-forth travel causes bleeding.' The Lancet Global Health has highlighted that rare disease patients globally face pervasive diagnostic delays and inadequate access to primary care services (28); the China Hemophilia Diagnosis and Treatment Report 2024 similarly confirms the reality of weak primary care capacity (27). These findings point to the construction of a ‘community–specialist linkage' referral system as a critical policy direction for reducing the cost of medical care and releasing patients' resources for social participation.

Social bias represents the most enduring macro-system influence on social participation. Cultural stigma systematically excludes patients from social participation through multiple channels, including matrimonial rejection, neighborhood gossip, and employer discrimination. The Lancet has specifically published work highlighting that rare disease stigmatization exists in virtually every country globally and systematically produces inequality, marginalization, and exclusion (29). A distinctive Chinese feature revealed by the present study is that grassroots discrimination against hemophilia is rooted in a cultural fear of ‘hereditary disease'—captured by P4′s statement that ‘if word gets out, you won't even be able to find a wife.' The essence of this exclusion is the avoidance of a perceived risk to family lineage continuity, rather than merely a reaction to the patient's current symptoms; its damage to social participation extends far beyond the employment discrimination described in Euro-American literature. A 2025 survey of Chinese hemophilia patients further confirms that stigma inflicts severe psychological and social impact, and that strengthening social support is a key pathway to expanding social participation (25).

From a nursing professional standpoint, macro-system transformation extends beyond the direct scope of individual clinical care; however, nursing professionals—as the professional group closest to patients' lived experiences of social participation difficulties—bear an irreplaceable role in policy advocacy and public education. Specifically, nursing professional organizations can: (1) collaborate with patient organizations to conduct social awareness campaigns that correct public misconceptions and biases about hemophilia, opening space for social participation from a cultural dimension; (2) participate in developing and refining hemophilia health insurance policies, advocating for a shift in coverage standards from uniform to severity-stratified, thereby institutionally guaranteeing the material prerequisites for patients' participation; and (3) advocate for an integrative care system centered on hemophilia treatment centers and extended through community health services, reducing the time and physical cost of medical care while releasing resources for social participation activities.

## Strengths and limitations

This study has several strengths. First, it is among the first to systematically apply social-ecological systems theory to the study of social participation barriers among people with hemophilia in China, providing a novel multi-level analytical framework for this under-researched population. Second, the use of purposive and snowball sampling strategies with maximum variation sampling ensured diversity in participant characteristics across age, disease severity, educational background, occupational status, and geographic origin, enhancing the breadth and depth of the findings. Third, multiple strategies to ensure trustworthiness—including member checking, independent dual-researcher coding, peer debriefing, and reflexive journalling—lend strength to the credibility of the findings.

Several limitations should be acknowledged. First, participants were recruited from a single tertiary hospital, and findings may not be fully transferable to patients in different institutional settings or geographical regions, particularly those in areas with even more limited healthcare resources. Second, all participants were male, reflecting the predominantly male epidemiology of hemophilia; this limits exploration of the potentially distinct social participation experiences of female carriers or affected females. Third, the study focused on patients' subjective experience and did not include the perspectives of family members, healthcare providers, or policymakers; future research incorporating multiple stakeholder perspectives would provide a more comprehensive understanding. Fourth, as a cross-sectional qualitative investigation, this study cannot capture dynamic changes in social participation barriers across the disease trajectory over time. Longitudinal designs would be valuable in future work. Fifth, the participants were recruited during hospitalization at a tertiary center and may have been in a “care-seeking” mindset, potentially over-reporting the difficulties they experienced; the interviewer's established rapport with participants and the use of open-ended, non-leading questions helped mitigate this risk.

## Conclusions

Social participation barriers among people with hemophilia are not a single-level problem but the result of interwoven, compounding barriers across micro-, meso-, and macro-system levels, with barriers at each level mutually reinforcing one another to generate cumulative deprivation of social participation. At the micro-system level, progressive joint damage and functional limitations, psychosocial distress and maladaptive coping, and inadequate disease knowledge and self-management deficits restricted patients' physical functioning, psychological well-being, and self-care capacity, thereby undermining their social participation. At the meso-system level, catastrophic medication costs and family resource depletion, family caregiving deficits and role overload, and employment exclusion and social network contraction drained family resources, intensified caregiving burden, and narrowed employment opportunities and social networks, further restricting social participation. At the macro-system level, insurance coverage gaps and treatment access barriers, geographic disparities in healthcare access, and social stigma and inadequate institutional support created structural obstacles that excluded patients from full and meaningful social participation. The nursing profession must transcend clinical boundaries, actively engaging in policy advocacy, public education, and multi-disciplinary collaboration with policymakers, patient organizations, and community stakeholders to address the structural barriers that prevent people with hemophilia from achieving full and meaningful social participation.

## Data Availability

The raw data supporting the conclusions of this article will be made available by the authors, without undue reservation.
